# Forensic Use of the Five Domains Model for Assessing Suffering in Cases of Animal Cruelty

**DOI:** 10.3390/ani8070101

**Published:** 2018-06-25

**Authors:** Rebecca A. Ledger, David J. Mellor

**Affiliations:** 1 Animal Welfare and Behaviour Consulting, P.O. Box 72012, Sasamat RPO, Vancouver, BC V6R 4P2, Canada; 2 Animal Welfare Science and Bioethics Centre, School of Veterinary Science, Massey University, Palmerston North 4474, New Zealand; d.j.mellor@massey.ac.nz

**Keywords:** abuse, cruelty, neglect, ill-treatment, expert opinions, persuasive arguments, sentience, negative affective experiences, suffering, Five Domains Model, canine examples, recommendations

## Abstract

**Simple Summary:**

Courts hearing cases about alleged ill-treatment of animals frequently utilise expert opinions which evaluate the nature and seriousness of reported negative welfare impacts. For several decades the Courts have required statements about such impacts to be supported mainly by physical and/or clinical evidence, usually regarding commentary about animals’ subjective experiences as being scientifically unsupported anthropomorphic speculation. This approach is well aligned with a view of animal welfare developed in the 1980s which emphasised scientifically validated features of “biological functioning” and, at an extreme, rejected any reference to subjective experiences that animals might have. However, subjective experiences, which include emotions, feelings, moods, and motivations, technically known as affects or affective states, became an increasing focus for animal behaviour scientists from the 1990s. This is now known as the “affective state” conceptual framework and it has been strengthened during the last two decades by integrating the findings of animal behaviour scientists and neuroscientists who explored brain processes that generate affective experiences. This provided cogent scientific support both for the existence of specific affects and the use of animals’ behaviour and some physiological responses to identify them. Two outcomes are noteworthy: first, that an animal’s welfare state is now very widely regarded by animal welfare scientists to reflect all of its affects experienced at any particular time, i.e., what the animal is experiencing subjectively; and second, that the extensive scientific understanding of the brain processes underlying affective experiences now convincingly negates spurious accusations of anthropomorphic speculation. These and other matters are considered here. It is concluded that the Courts’ current heavy reliance on physical and/or clinical evidence of ill-treatment should be modified. Instead, Courts should now recognise—as validly based—expert opinions that provide cogent evidence of untoward affective outcomes caused by ill-treatment, supplemented where appropriate by any relevant physical and/or clinical evidence if that is available.

**Abstract:**

Conceptual frameworks for understanding animal welfare scientifically are widely influential. An early “biological functioning” framework still influences expert opinions prepared for Courts hearing animal cruelty cases, despite deficiencies in it being revealed by the later emergence and wide scientific adoption of an “affective state” framework. According to “biological functioning” precepts, indices of negative welfare states should predominantly be physical and/or clinical and any that refer to animals’ supposed subjective experiences, i.e., their “affective states”, should be excluded. However, “affective state” precepts, which have secure affective neuroscience and aligned animal behaviour science foundations, show that behavioural indices may be utilised to credibly identify negative welfare outcomes in terms of negative subjective experiences, or affects. It is noted that the now very wide scientific acceptance of the “affective state” framework is entirely consistent with the current extensive international recognition that animals of welfare significance are “sentient” beings. A long list of negative affects is discussed and each one is described as a prelude to updating the concept of “suffering” or “distress”, often referred to in animal welfare legislation and prosecutions for alleged ill-treatment of animals. The Five Domains Model for assessing and grading animal welfare compromise is then discussed, highlighting that it incorporates a coherent amalgamation of “biological functioning” and “affective state” precepts into its operational features. That is followed by examples of severe-to-very-severe ill-treatment of dogs. These include inescapable psychological and/or physical abuse or mistreatment, excessively restrictive or otherwise detrimental housing or holding conditions, and/or seriously inadequate provision of the necessities of life, in each case drawing attention to specific affects that such ill-treatment generates. It is concluded that experts should frame their opinions in ways that include negative affective outcomes. Moreover, the cogency of such analyses should be drawn to the attention of the Judiciary when they are deliberating on suffering in animals, thereby providing a basis for them to move from a current heavy reliance on physical and/or clinical indices of cruelty or neglect towards including in their decisions careful evaluations of animals’ negative affective experiences.

## 1. Introduction

Understanding evolves. This is as true for animal welfare science [1,2,3,4,5,6] as it is for every other scientific discipline. Understanding of what compromised and good animal welfare represent is particularly relevant in the context of prosecutions for animal cruelty understood to include abuse and neglect. This understanding provides the foundations upon which credible evidence of the ill-treatment of animals is assembled and tested. This evidence is marshalled by using the specific indices of poor welfare that are regarded as valid at the time the prosecutions are pursued. During the last 30 years, however, there have been major shifts in the conceptual frameworks or scientific orientations that underpinned animal welfare understanding and assessment [2,3,5,7]. It is therefore helpful to consider these changes in order to clarify how new indices of poor welfare status emerged, were validated, and now enable the negative impacts of ill-treatment to be evaluated in greater depth than was possible previously.

This paper begins by describing the shifts in understanding that are already changing the focus of expert opinions concerned with cruelty towards animals. Specifically, it outlines the science-based rationale for extending the current strong emphasis on physical and/or clinical signs of ill-treatment to also include negative subjective experiences that sentient animals are understood to have. Next, these negative experiences are listed, described, and discussed, and brief reference is also made to positive subjective experiences. The concept of “suffering” or “distress” is then updated in view of its implicit or explicit role in most prosecutions for ill-treatment. This is followed by a description of the Five Domains Model and its use to facilitate thorough forensic examination of animal welfare compromise. Then, using dogs to illustrate welfare outcomes, some specific forms of severe-to-very-severe cruelty and neglect and related negative subjective experiences are described. The paper ends with recommendations regarding a fresh approach to formulating expert opinions prepared for Courts that hear cruelty cases.

## 2. Changes in Conceptual Frameworks for Understanding Animal Welfare

Three key conceptual frameworks or orientations which influenced scientific investigations and commentaries that focused on understanding and managing animal welfare emerged in the 1980s and 1990s. The appellations assigned to them were “biological functioning”, “affective state”, and “natural living” [7]. The first two are especially relevant to contemporary forensic assessment of poor animal welfare states because they deal with the interactive functionality within the body and the subjective experiences that animals may have. The last of the three, “natural living”, which focuses mainly on the external circumstances of animals, is relevant only to the extent that those circumstances can have major impacts on internal functionality and subjective experiences, which, as just noted, are more directly pertinent to understanding animal welfare [5,6,8].

### 2.1. Emergence of Two Key Conceptual Frameworks: Biological Functioning and Affective State

In the 1980s a biological functioning framework or orientation was emphasised. According to this an animal would have good welfare when, among other attributes, it grew well, was in good health, reproduced successfully, and was relatively stress free [4,7,9,10]. This orientation emphasised the physical wellbeing of animals such that physical or clinical indices of poor growth, health, and reproductive outcome and/or physiological indices of the presence of stress appeared to be a sound basis for demonstrating welfare compromise. At that time, scientists were strongly discouraged from inferring that animals could experience subjective mental states such as emotions, feelings, moods, or motivations, collectively known as affects or affective states [3,11,12,13,14]. This was because such experiences were considered to be anthropomorphic speculations that lacked scientific support. However, by de-emphasising relationships between physical wellbeing and mental states, the perspectives provided by the biological functioning orientation, although useful, were limited. 

In the early 1990s other animal welfare scientists gave increasing attention to the mental experiences that animals could have, including both negative and positive affective states [7,12,15,16]. It took until the mid-1990s before this approach began to be accepted scientifically. By the early 2000s, however, focusing on affective states was well recognised [12,17] and subsequently became a key element of much animal welfare science thinking (e.g., [3,4,5,18,19]). Confidence in this orientation was bolstered by substantial developments in affective neuroscience, i.e., the discipline concerned with understanding the brain processes that generate the negative and positive affects animals are understood to experience (e.g., [13,19,20,21,22,23]).

According to the affective state orientation, an animal’s welfare would be good when it responds with positive affective experiences during its interactions with other animals, people, and the environment, and with few negative affective experiences [10]. Indices of specific affects, derived with affective neuroscience support (e.g., [13,24,25,26,27,28,29,30,31,32,33,34]), are predominantly behavioural. They have been clarified mainly by behavioural science investigations of animals’ preferences, aversions, and priorities (e.g., [35,36,37]), and also by observing their natural behaviours (e.g., [3,35,38,39,40,41,42,43,44,45,46,47,48,49,50]). 

It is important to note that interpretation of behavioural indices of particular affective experiences is closely linked to the circumstances of the animal. Understanding these links enables conclusions to be drawn about the affects animals are most likely to have experienced in two ways: first, when indicative behaviours are observed directly (e.g., very loud, urgent vocalisations); and second, when the only thing that is known are the circumstances that give rise to particular indicative behaviours (e.g., being thrown into a fire or boiling water). In other words, identifying the likely affective outcomes of particular situations can be approached by emphasising *observed behaviours* or by focusing on the *known circumstances* of the animals, or both (see Section 6). 

### 2.2. Integrating the Conceptual Frameworks of Biological Functioning and Affective State

For some years the proponents of the biological functioning and affective state orientations considered them to be competing conceptual frameworks for understanding animal welfare. During the last decade, however, animal welfare scientists have come to accept that both are relevant to individual animals living dynamically as whole biological entities [3,4,5,6,19,23,49,50,51]. It is apposite to note here that this dynamically integrated understanding was incorporated into the rationale of the Five Domains Model, referred to in Section 5, from its inception in 1994 [6,52]. Thus, biological functioning underlies affective experience and affective experience influences biological functioning, so that validated indices of both are now well understood to be informative with regard to different aspects of animal welfare compromise and enhancement [49,51,53]. These developments in thinking have contributed to the now very wide acceptance that an animal’s welfare is directly related to what it experiences subjectively, i.e., its affective experiences, whether they are negative or positive (see [6,51,53].

### 2.3. Expert Opinions and Conceptual Frameworks for Understanding Animal Welfare

Most past and present expert opinions reflect a strong leaning towards the biological functioning framework of animal welfare science thinking. Aligned with this, the Courts have long regarded physical and/or clinical evidence of ill-treatment and related suffering or distress to be more persuasive than assertions about the negative affective experiences animals may have. To date, therefore, the prospect of Judges and/or Defence Counsel challenging inferences about affects as unsubstantiated anthropomorphic speculation has led to virtually all references to affects being excluded from most expert opinions, notably, apart from “pain”.

The recently demonstrated alignment of affective neuroscience and animal behaviour science has changed this situation. In particular, this alignment negates assertions of unsubstantiated anthropomorphism by demonstrating that specific brain processes are associated with the generation of particular affects. Thus, affective neuroscience has provided compelling evidence that a wide range of negative and positive affects are indeed experienced by mammals and birds (e.g., [2,5,6,20,23,49,54,55]). Moreover, taking into consideration the specific circumstances of animals whose welfare is being assessed, this alignment has also validated the use of some behavioural indices to confidently assert that particular affects are present or absent. These indices include an animal’s observed activity/inactivity, vocalisation/silence, demeanour, and appearance (e.g., [3,35,36,37,48,49,50,51]). 

In light of this, there is an apparent need to revise the main conceptual framework utilised in expert opinions, to refocus it onto what for some years now has been regarded as the *raison d’être* of animal welfare understanding; namely, the capacity of animals to have negative and positive affective experiences and what particular affects are experienced in the many different external circumstances animals encounter. 

### 2.4. Expert Opinions That Incorporate Consideration of Affective States: Canadian Experience

Expert opinions prepared by the first author (R.A. Ledger) have included details of negative affective states that indicate dogs subjected to ill-treatment had suffered. These opinions were well received by Canadian Courts which found the evidence presented in this way persuasive [56]. Expert opinions of this type have been applied in at least 32 cases across Canada since 2014 and have resulted in warrants to seize the animal(s) being approved or charges being laid in 31 of these cases. Of these, the accused was found guilty of causing an animal or animals unnecessary suffering in 15 cases, 6 cases are still awaiting trial, and in the remaining 10 cases, charges were dropped for reasons unrelated to the affective state content of the expert opinions (R.A. Ledger, unpublished records).

There are notable examples of animal cruelty prosecutions that succeeded where affective state information was presented and physical evidence was not available, and others where the affective state information complemented existing physical evidence that would otherwise have been inadequate. Three cases are presented below.

*Case 1, Regina vs Paulsen (2015)* [57]: The accused left 6 dogs in a parked vehicle in air temperatures of about 27 °C. All 6 dogs died from hyperthermia. The Prosecution argued that all of the dogs would have experienced significant emotional suffering and distress as a direct result of the heat in the enclosed canopy of the pickup truck—specifically, anxiety, panic, nausea, and thermal and physical discomfort. This affective analysis was considered as fact in the Court’s decision. In finding Paulsen guilty of causing all 6 dogs unnecessary suffering, the nature and manner in which the dogs died was considered an aggravating factor in Paulsen’s sentencing, which included a 6-month prison sentence.

*Case 2, Regina vs Hague (2015)* [58]: The accused was observed inside an elevator, kicking a Doberman puppy and jerking her by the leash. The British Columbia Society for the Prevention of Cruelty to Animals (BC SPCA) seized the dog, but a detailed examination revealed no physical signs of abuse. The case proceeded based on the circumstances of the incident (being kicked and jerked) and the behavioural response of the dog, which indicated she experienced fear and pain during the abusive act. The accused pled guilty to causing an animal unnecessary emotional distress and was sentenced to a $5000 fine and a 3 years prohibition order.

*Case 3, BC SPCA vs Viitre (2016)* [59]: The accused was observed leaving his German shepherd dog confined inside a vehicle for prolonged periods, and of striking the dog harshly across the head. The BC SPCA seized the dog from the accused. A detailed examination by a veterinarian determined that the dog had no signs of physical injury. The accused subsequently appealed the BC SPCA’s decision to seize his dog, requesting that his dog be returned to him. The Farm Industry Review Board upheld the BC SPCA’s decision, denying the accused the return of his dog, citing the negative emotional impact that this would likely have on the dog.

Generic examples of evidence related to severe-to-very-severe cruelty or neglect, unrelated to specific court cases, are provided in Section 6.

### 2.5. Sentience and Up-To-Date Animal Welfare Understanding Are Well Aligned

Sentience is the capacity of animals to perceive by the senses and, thereby, to consciously experience both negative and positive affects which are important to them and which influence their welfare. Thus, the above welfare-related understanding clearly refers to sentient animals. It is worth noting that for at least 15–20 years there has been rising international recognition that animals whose welfare is of interest are sentient. More recent declarations to this effect have been made in the European Union via the Treaty of Lisbon in 2008 [60] and via laws enacted in 2015 in France [61], New Zealand [62], and Quebec [63]. In addition, the same principle of animal sentience was affirmed by at least 46 countries which supported a 2014 proposal that the United Nations should issue a Universal Declaration on Animal Welfare [64]. Finally, the 180 member countries of the World Organisation for Animal Health (OIE), in adopting the OIE Global Animal Welfare Strategy 2017, also accepted a statement recognising animal sentience [65]. Accordingly, brief reference in expert opinions to these international developments could add to the persuasiveness of opinions that emphasise affects.

## 3. Negative and Positive Affects That Sentient Animals Are Considered to Experience

In a detailed, fully referenced characterisation of what animal welfare is currently considered to represent, Mellor [6] drew attention to key features of the negative and positive affects animals may experience in the following way, now quoted verbatim:

- There are two major types of negative affective experiences: those that reflect imbalances or disturbances in the internal physical/functional state of the body, and those elicited from outside the body that contribute to an animal’s perception of its external circumstances.
- Internally generated negative affects include breathlessness, thirst, hunger, pain, nausea, dizziness, debility, weakness, and sickness. Each of these affects motivates animals to behave in particular ways that help to secure their survival. However, correction of the associated imbalance or disturbance, whether achieved by the animal unaided or with support from animal care staff, at best will usually only result in a neutral, not positive, affective outcome.
- Externally generated negative affects include anxiety, fear, panic, frustration, anger, helplessness, loneliness, boredom, and depression. These are mainly elicited by threatening, cramped, barren, and/or isolated circumstances, and will persist for as long as such conditions prevail. These are situation-related negative affects and human intervention is usually required to correct them.
- Providing animals with opportunities to engage in behaviours they find rewarding can replace situation-related negative affects with positive experiences. Such opportunities become available when social animals are kept with congenial others in spacious, stimulus-rich, and safe environments.
- Rewarding behaviours may arise when the key attributes of animals’ environments include, but are not limited to, the following: variability that provides a congenial balance between predictability and unpredictability; access to preferred sites for resting, thermal comfort, and voiding excrement; environmental choices that encourage exploratory and food acquisition behaviours which are enjoyable; availability of a variety of feeds having pleasurable tastes and textures; and circumstances that enable social species to engage in bonding and bond affirming activities and, as appropriate, other affiliative interactions such as maternal, paternal, or group care of young, play behaviour, and sexual activity. Expressed in general terms, the associated positive affects are likely to include various forms of comfort, pleasure, interest, confidence, and a sense of control.

In addition, the negative affects generated by internal physical/functional imbalances or disruptions apparently interact with the motivation of animals to engage in rewarding behaviour. Thus, when the intensity of such negative affects is marked, animals usually do not engage in rewarding behaviours even when opportunities to do so are available [6,49,53,66]. For example, such demotivation is apparent in animals experiencing severe breathlessness, nausea, pain, sickness, or weakness.

The above understanding is obviously directly relevant to the framing of expert opinions which conclude that actionable cruelty or neglect has occurred, especially where the conclusions are based mainly on scientifically supported evaluations of the negative affects the animals in question are likely to have experienced. Table 1 provides a brief description of what each negative affect is understood to represent.

Examples of severe-to-very-severe impositions that would undoubtedly lead to significantly negative affective outcomes include inescapable psychological and/or physical abuse or mistreatment, excessively restrictive or otherwise detrimental housing or holding conditions, and/or seriously inadequate provision of the necessities of life (see Section 6). As the affective outcomes of such ill-treatment may be described generically in terms of “suffering” or “distress”, it would be helpful to briefly consider what these generic terms are now understood to represent.

## 4. Updated Understanding of the Concept of Suffering or Distress

As generic terms, suffering and distress are equivalent. Suffering is a term encompassing unpleasant, undesired states of being which are the outcome of the impact on an animal of one or more of a variety of noxious stimuli, often accompanied by the absence of important positive stimuli [4,17]. Suffering is the opposite of good welfare. It may manifest as very aversive physical, mental, and/or emotional experiences, including unpleasant feelings, sensations, or perceptions, cognitively processed and interpreted by the animal according to it species-specific and individual nature, and past experience. 

There are numerous negative subjective affects that animals are likely to experience where the impact of their character, intensity, and/or duration can be sufficiently aversive or extreme for them to be described in terms of suffering. These affects include many types of pain, as well as thirst, hunger, debility, breathlessness, nausea, sickness, anxiety, fear, panic, nervous vigilance, loneliness, helplessness, frustration, and anger, and other as-yet-unspecified forms of distress [4,16,17,49,55,67]. However, note that when specific negative affects approach their extreme, they are not transformed into an experience of “suffering”; rather, they retain their original character so that, for example, intense breathlessness continues to be experienced as breathlessness. The same is true for thirst, hunger, pain, nausea, anxiety, fear, panic, or depression, as these and all other such negative experiences also retain their individual character when they are present at high intensities. Yet it is possible for two or more such affects to be present at the same time when conditions that give rise them exist simultaneously, although if one of them, such as pain or breathlessness, is much more intense than, say, thirst or hunger, the more intense experiences may largely or even completely dominate the animal’s awareness. It is also possible that the negative impact of one affect may generate one or more other strongly negative experiences: for example, in human beings when breathlessness at its extreme brings with it concern about imminent death, this can lead the person to experience severe fear and/or panic as well [55]. Likewise, frustration can also be associated with feelings of anxiety [68] and anger [69].

## 5. The Five Domains Model for Animal Welfare Assessment

Originally formulated in 1994 to identify and grade negative impacts of research, teaching, and testing procedures involving sentient animals [52], the Five Domains Model (hereafter referred to as “the Model”) has been used as a formal part of New Zealand’s regulatory approval process for the conduct of animal-based science since 1997 [70]. During the intervening period the Model has been regularly updated and extended to incorporate, at each stage, the latest validated scientific knowledge relevant to animal welfare [4,5,49,53,70,71,72,73,74,75]. In this way the list of specified negative affective experiences was extended in order to give greater definition to the generic notions of suffering and distress (see Table 1). Although of less value for scrutinising situations that potentially involve cruelty or neglect, a more recent update incorporated a list of positive affective experiences of relevance to animal welfare enhancement [49]. Other updates extended Model applications to animals used or managed in wider contexts, e.g., to farm livestock, draught animals, working dogs, companion pets, sport and recreation animals, service animals, zoo and free-living wildlife, and pest animals [4,55,66,67,71,76,77,78,79,80,81,82,83]. 

Of relevance to Section 6 below, Littlewood and Mellor [66] used a fictitious scenario involving a working farm dog to demonstrate how the Five Domains Model may be used. The scenario described sequential welfare assessments in a farm dog before, during, and after it became entangled in barbed wire, which required a leg amputation and rehoming to a lifestyle hobby farm. This example, in common with others [4,67,71,76,77,78,81,82], shows how the Model was designed to facilitate the assessment and grading of animal welfare impacts in a systematic, structured, comprehensive, and coherent manner. It is important to note that published details of how the Model may be used to assess and grade animal welfare compromise are available for cost-free downloading from *Animals* [53,66].

**Figure 1:**
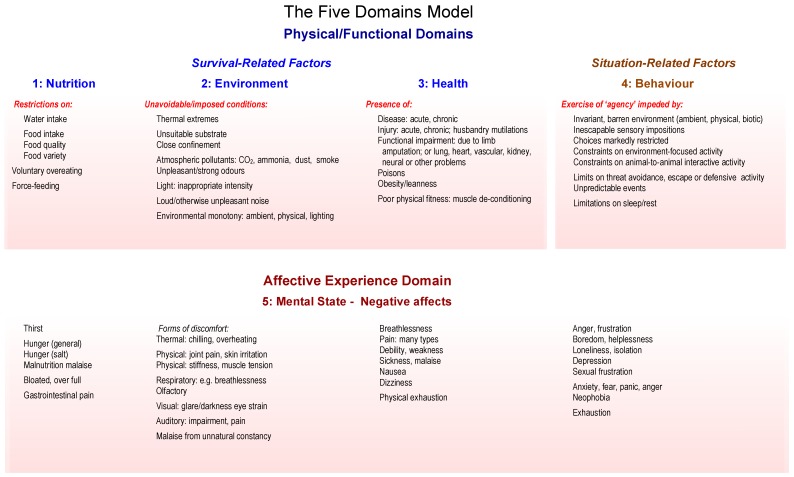
A version of the Five Domains Model adapted from the most recent one [49,53] by removing all reference to welfare-enhancing factors and associated affects in order to make it more directly relevant to formulating expert opinions concerned with ill-treatment. The examples provided for the physical/functional Domains 1 to 3, labelled “Nutrition”, “Environment”, and “Health”, are intended to direct attention towards mainly internal *survival-related factors*, and those provided for Domain 4, labelled “Behaviour”, focus attention largely on external *situation-related factors* that affect the animal’s perception of its external circumstances. For each of Domains 1 to 4, examples of negative factors are provided and are aligned with inferred affective experiences, assigned to Domain 5, labelled “Mental State”. Note that an animal exercises “agency” (Domain 4: “Behaviour”) when it engages in voluntary, self-generated, and goal-directed behaviours [11,84]. A detailed explanation of how to operate the Model is available for downloading at no cost from *Animals* [53].

Thus, internal states and external circumstances of animals are evaluated thoroughly by referring to each of the first four physical/functional domains of the Model, designated “Nutrition”, “Environment”, “Health”, and “Behaviour”. The affective experiences cautiously inferred to be generated by factors identified in these four domains are then assigned to the fifth domain, designated “Mental State”. The purpose of each of the five domains, therefore, is to draw attention to many specific factors that underlie thorough animal welfare assessments. Of course, with regard to serious cases of suspected cruelty or neglect (see Section 6), the primary focus of such assessments is on the generation and presence of negative affective experiences, not positive ones. This is because in such cases the intensity of the negative affects would demotivate the animals from utilising existing opportunities to engage in rewarding behaviours [49,53]. Finally, note that a major role of the Model is to guide systematic, structured, comprehensive, and coherent identification of particular negative affects which are likely to be generated by different forms of ill-treatment. This information is then utilised during the framing of expert opinions. Figure 1 is available as an *aide memoire* to facilitate this process.

## 6. Examples of Cruelty or Neglect Interpreted by Reference to Affective Experiences in Dogs

Interactions between dogs and people are diverse and pervade numerous aspects of daily life [85,86]. They are kept for companionship as pets, as surrogate children, or to educate children in responsible care of a live dependent. They support people with impaired sight, hearing, or mobility. They are used to detect cancer or imminent epileptic seizures. They also have therapeutic benefits through visits to hospital patients and residents in rest homes and hospices. They are the focus of staged performances, films, dog shows, and agility competitions. Dogs are used to guard people, homes, and commercial premises; to track and subdue fugitives; to detect mines, drugs, or prohibited food stuffs; and in disasters such as earthquakes, to locate survivors in the rubble. Dogs are used to manage livestock on farms or to protect them from predators, and as draft animals to pull carts or sleds. They are also used in hunting, both for locating target animals and for retrieving shot birds, and domestic dogs that have become feral are themselves hunted. Dogs are the focus of the commercial greyhound racing industry. They are used in some animal-based scientific research. And providing dogs for virtually all of these activities are dog breeders, many of which are aligned with canine breed associations and societies.

A major part of the appeal of dogs is their impressive capacity to communicate with people [50,85,86,87,88], which undoubtedly also contributes to their ability to perform in such diverse roles. Accordingly, there is wide familiarity with expressive canine behaviours so that dogs are well suited to illustrating behavioural indices of negative affective experiences. Unfortunately, the diversity of their uses means that they also provide numerous examples of ill-treatment. Hence the present focus on dogs.

Table 2 contains examples of inescapable psychological and/or physical abuse or mistreatment, excessively restrictive or otherwise detrimental housing or holding conditions, and/or seriously inadequate provision of the necessities of life, all of which would lead to severe-to-very-severe-intensity negative affective experiences. Such subjective experiences may exhibit sequences that progress from initial to subsequent affects. For example, while in a hyperthermic state, an animal will initially experience the affect of thermal discomfort. As the animal attempts to cool itself through panting, without access to water it will eventually become dehydrated which may subsequently lead to thirst and nausea. Moreover, prolonged dehydration may cause organ failure and lead the animal to also experience pain. Also illustrative of such sequences are some of the anticipated post-traumatic emotional and behavioural consequences of cruelty to dogs, including an elevated incidence of anxiety and fear-related disorders [89].

Note that Table 2 provides generic examples of particular situations and related affective experiences derived from cases of severe-to-very-severe ill-treatment brought before Canadian and New Zealand Courts. Also note that some entries in Table 2 may represent an individual facet of cases that involve multiple acts of ill-treatment. In those cases, all facets would need to be evaluated. The examples provided are designed to facilitate that process. To illustrate, some brutal training methods may involve various combinations of suffocation, strangulation, and/or inflicting injuries in dogs that are intended to survive, and there are cases of abandonment which are preceded by the brutal infliction of major injuries. The examples, therefore, have not been assigned to rigid categories.

The list of acts of ill-treatment in Table 2 is not complete. Other such acts are numerous and include but are not limited to internal or external application of toxic or corrosive chemicals; inserting exploding fireworks into the mouth, anus, or vagina, exploding fireworks nearby, or aiming rockets directly at a dog; sadistic use of electric collars; penetrative genital wounding; and brutal treatment of puppies and lactating bitches. 

The majority of examples in Table 2 were chosen because the severe-to-very-severe nature of the abuse means that, in each case, there is virtually no doubt about which negative affect, or combination of affects, would have been experienced by the dogs. This level of severity is near to or at the worst level as judged using the Model’s 5-tier qualitative scale of “none”, “minor”, “moderate”, “severe”, and “very severe” negative welfare impacts [49]. These examples therefore provide a benchmark by which confidence in inferred affective outcomes of somewhat less severe, but still marked ill-treatment may be judged. 

## 7. Recommendations

It is recommended that the analysis outlined here be used to encourage animal welfare scientists, behaviour consultants, animal welfare inspectors, veterinarians, and others who prepare expert opinions to frame them in ways that include negative affective outcomes. During this early introductory stage, however, prudent selection of cases where the evidence is compelling is strongly recommended, as is great care in preparing opinions. This is because poorly argued, overstated, or otherwise unconvincing justification of conclusions based on affective outcomes could lead to judgements that hinder the wider adoption of this approach by the Courts. 

It is likewise recommended that the cogency of such analyses be drawn to the attention of cruelty investigators, Judges, Prosecutors, and Defense Counsel to help them move from a current heavy reliance on physical and/or clinical indices of cruelty or neglect towards including in their deliberations careful evaluations of animals’ negative affective experiences. 

Although this paper focuses largely on prosecutions involving severe-to-very-severe levels of suffering in animals, both Case 2, Regina vs Hague (2015) [58], and Case 3, BC SPCA vs Viitre (2016) [59], described in Section 2.4, involved suffering that may be considered moderate to severe. In British Columbia, Canada, where these cases were heard, the prosecution is merely required to prove that an animal has suffered, and not to demonstrate the degree to which it has suffered. As such, whilst cruelty prosecutions may proceed most confidently in cases of severe-to-very-severe suffering, other less heinous cases may still be considered relevant. The law does refer, however, to the doctrine of *de minimis non curat lex*, by which the law does not consider trivial matters. This means that mild forms of suffering in animals would typically not be considered relevant under the law.

On the basis of the first author’s direct experience, it is recommended that a sequence of eight questions be followed when expert opinions are being prepared:

*1.*  Can the animal experience aversive events that could cause it to suffer?
  If the animal is sentient and was/is conscious when ill-treated, the answer may be “Yes” (see Section 2.2, Section 2.3, Section 2.4 and Section 2.5). The ability of an animal to experience various negative affects will vary between individuals, according to age, species, previous experience, and personality.
*2.*  Does current legislation recognise suffering or equivalent negative welfare states in animals?
  The precise wording of national or regional laws or regulations in relation to the up-to-date understanding of suffering and related matters outlined in Section 4 should indicate what would be credible grounds for a prosecution in each country. Expert opinions need to be framed with this in mind, and, of course, experts must first evaluate whether a prosecution can be mounted at all given the particularities of the relevant laws or regulations.
*3.*  Were conditions present that would cause the animal to suffer?
  Systematic evaluation of circumstances related to the nutritional, environmental, health, and behavioural domains of the Model will assist here (see Section 5). It is helpful to use a checklist for recording factual information about each domain. The checklists developed as part of a protocol strongly focused on suffering understood generically [90], which does not incorporate the detailed affective evaluations described here, would nevertheless be a good starting point.
*4.*  Which affective state(s) would the animal likely experience?
  Reference to the Model helps when answering this question (see Section 5).
*5.*  Is there physical and/or behavioural evidence that the animal actually did suffer or is suffering?
  This may be evaluated by direct observation of the animal supported by careful interpretation of other specific elements of the evidence including the animal’s precise circumstances (Section 6: e.g., Table 2), during and/or following the abusive or neglectful act. Use of detailed checklists such as those mentioned above [90] would be helpful.
  However, it is important to carefully consider possible alternative explanations for the occurrence of the observed physical and behavioural responses of the animal in the circumstances under review. For example, in the case of an animal that shows physiological and/or behavioural signs of fear, causes other than the circumstances imposed by the accused should also be considered.
*6.*  How severe and protracted was or is the suffering?
  This may be evaluated qualitatively by carefully interpreting the circumstances and the physical and/or behavioural evidence (e.g., Table 2), guided by using the Model’s 5-tier negative impact scale [49,53].
*7.*  Could the suffering have been avoided? Was it necessary?
  This refers to situations where there might have been reason to believe that the negative impacts were justified. Subsidiary questions that help to clarify these points are the following: *Was a more humane option available? Could the overall benefit to the animal justify the negative impact of what was done?*
*8.*  Was the suffering inflicted willfully or recklessly?
  This question helps to distinguish purposeful cruelty from indifferent disregard, stupidity, ignorance, and naïveté, e.g., situations where the alleged perpetrator might not have anticipated the negative welfare impacts, or the possibility that they could occur.

Finally, the examples of abuse and neglect considered here refer only to dogs. It is recommended, therefore, that these examples be used to guide careful evaluations of the welfare consequences of ill-treatment in other species, taking into consideration what is known about species-specific sensory capacities and behaviour expressed through known interactions of these animals with their normal physical, biotic, and social environments and their known responses to imposed environments. Of course, what is actually known about such attributes might variously be extensive or limited, depending on the species; hence, caution is recommended.

## Figures and Tables

**Table 1:** Details of negative affective experiences generated within the body and others that reflect an animal’s perception of its external circumstances.

**Negative Affective Experiences Generated within the Body**	
Breathlessness	An urgent compulsion to increase respiratory activity (e.g., breathing rate and depth; gasping), to overcome resistance to airflow due to obstructions in air passages (e.g., laboured breathing), or to escape from external impediments to breathing; can lead to anxiety, fear, and panic
Thirst	A compulsion to seek and drink water
Hunger	A compulsion to seek and eat energy-rich and other foods
Pain	Noxious experiences associated with physical injury or the threat of such injury
Nausea	A sensation of unease and discomfort in the upper stomach linked to an involuntary urge to vomit; it may precede vomiting, but can occur without it
Dizziness	Impaired spatial perception and stability; feelings of disequilibrium or giddiness
Weakness	A negative feeling associated with reduced strength, muscle tone, vigour, or fitness
Exhaustion	A negative feeling associated with physical and/or mental fatigue linked to excessive metabolic demands (e.g., sustained strenuous exercise, repeated pregnancy/lactation) and/or persistently inadequate sleep/rest
Debility	A negative feeling associated with physical weakness, especially associated with illness
Sickness	Feelings of lethargy, depression, sleepiness, and reduced appetite associated with fever; can be associated with seeking isolation or comforting social contact
Physical discomfort	Feelings of irritation such as itching from dust or allergens, joint stiffness due to cramped space, and/or the unpleasantness of being on hard or rough surfaces
Thermal discomfort (too cold)	Distress of chilling caused by marked-to-severe cold, draughty, and/or wet conditions; severe cold may induce pain; when hypothermic cannot get or stay warm
Thermal discomfort (too hot)	Distress of overheating caused by marked-to-severe radiant and/or ambient heat; radiant heat can lead to pain; when hyperthermic cannot get or stay cool; may progress to dizziness, fainting, weakness, and/or pain associated with organ shutdown
Auditory discomfort	Distress experienced when the volume, pitch, and/or rhythm of sound is uncomfortable; may lead to pain and hearing impairment
Olfactory discomfort	Distress experienced in the presence of unpleasant odours and/or irritants, e.g., smoke, urinary ammonia, fecal smells, dust, and carbon dioxide
Visual discomfort	Distress experienced in the presence of light conditions that create eye strain, including dark, dim, and glaring light; can lead to pain
**Negative Affective Experiences that Reflect an Animal’s Perception of its External Circumstances**	
Anxiety	A negative feeling experienced in anticipation of a threat to safety
Fear	A negative feeling experienced during a perceived threat to safety
Panic	A sudden, uncontrollable, and intense level of anxiety or fear
Frustration (general)	Exasperation at being delayed or thwarted in achieving an internally or externally motivated goal; can lead to anxiety and/or anger
Frustration (sexual)	Exasperation at being delayed or thwarted in engaging in desired sexual experiences
Anger	An intense emotion usually involving agitation and a strongly hostile response to a perceived provocation, challenge, or threat
Helplessness	A negative feeling associated with an inability to cope or act effectively, due to repeated unsuccessful attempts to do so
Loneliness	A negative feeling related to a lack of connection or communication with others
Boredom	A specific mental state where a lack of stimulation leads to craving for relief
Depression	A state of feeling sad, hopeless, helpless, and dejected, accompanied by a reduced ability to have pleasurable experiences
Malaise	A vague or unfocused feeling of lethargy, weakness, or discomfort associated with unremitting constancy in barren environments

**Table 2:** Examples of cruelty and neglect towards dogs. The description of each act of cruelty includes brief examples of specific situations, the likely behavioural responses, and the negative affective experiences inferred from the situation and/or behavioural responses. See Table 1 for a brief description of each affective experience. Note that when all the examples provided are judged on a 5-tier scale of “none”, “minor”, “moderate”, “severe”, and “very severe” acts of cruelty or neglect [49], the majority fall into the severe-to-very severe categories, where the occurrence of negative affects would not be in doubt. * denotes situations that may result in death.

Act of Cruelty or Neglect	Specific Situations	Behavioural Responses	Inferred Affective Experiences
* Suffocation: impeding breathing to kill or torture	Sealing the head or whole body in a plastic bag; blocking the nose and mouth by hand	Extreme struggling and escape attempts, accompanied by gasping Survivors may cower, withdraw, or show appeasement gestures in circumstances similar to those in which the cruel act took place	Extreme breathlessness accompanied by anxiety, fear, and panic, and physical exhaustion Anxiety and fear may be expressed by survivors in circumstances similar to those in which the cruel act took place, e.g., presence of the abuser, location where the abuse took place, exposure to equipment associated with the cruel act
* Drowning: the use of water to impede breathing to kill, torture, or punish	Submerging in deep or turbulent water; restraining the animal under running water; hosing the face with water	Extreme struggling and escape attempts, accompanied by gasping Survivors may cower, withdraw, or show appeasement gestures in circumstances similar to those in which the cruel act took place	Extreme breathlessness accompanied by anxiety, fear, and panic, and physical exhaustion Anxiety and fear may be expressed by survivors in circumstances similar to those in which the cruel act took place, e.g., presence of the abuser, location where the abuse took place, exposure to equipment associated with the cruel act
* Strangulation: obstructing breathing to kill or punish	Compressing the trachea by hand; suspending or forcefully swinging the dog by a neck rope or collar	Extreme struggling accompanied by gasping; attempts to vocalise; body may become limp Survivors may cower, withdraw ,or show appeasement gestures in circumstances similar to those in which the cruel act took place	Extreme breathlessness, anxiety, fear, and panic, accompanied by pain from tight neck ligature; physical exhaustion. Anxiety and fear may be expressed by survivors in circumstances similar to those in which the cruel act took place, e.g., presence of the abuser, location where the abuse took place, exposure to equipment associated with the cruel act
* Injuring: inflicting blunt force trauma to punish or torture	Severe beating with fist, club, whip, or other solid object; kicking; throwing or swinging against solid objects	Loud distress calls, escape attempts, cowering; snarling or trying to attack abuser; subsequent guarding injured areas, reduced mobility, withdrawal, and vocalisations when manipulated, showing appeasing behaviours Survivors may cower, withdraw, or show appeasement gestures in circumstances similar to those in which the cruel act took place	Extreme pain, fear, and panic during physical assaults; persistent subsequent marked pain from fractures and other serious injuries Anxiety and fear may be expressed by survivors in circumstances similar to those in which the cruel act took place, e.g., presence of the abuser, location where the abuse took place, exposure to equipment associated with the cruel act
* Shooting:	Shooting by bullet, pellet, arrow, or nail	If not immediately fatal, responses depend on effects of wound site(s) on the capacity to escape	Moderate-to-extreme pain, anxiety, and fear; fear extreme when escape is hindered by bone fractures, paralysis, blindness, or brain damage
* Vehicular abuse:	Being dragged behind, thrown from, or run over by a vehicle	Loud distress calls, struggling, escape attempts, guarding injured areas, impaired mobility or paralysis	Extreme fear and panic plus pain during each type of assault and breathlessness if dragged by the neck; then persistent pain from fractures, areas excoriated to the bone, and other external or internal injuries, as well as fear and helplessness
* Burning or boiling alive:	Applying accelerant and lighting it; throwing dog into a fire or boiling water; microwaving	Loud distress calls, vigorous escape attempts, writhing until death. Survivors withdrawn, immobile, vocalise when moved; may cower, withdraw, or show appeasement gestures in circumstances similar to those in which the cruel act took place	Extreme pain, fear, and panic until death; in survivors, persistent marked-to-extreme pain and fear, plus extreme weakness Anxiety and fear may be expressed by survivors in circumstances similar to those in which the cruel act took place, e.g., presence of the abuser, location where the abuse took place, exposure to equipment associated with the cruel act
* Dog fights:	Pitting aggressive dogs against other aggressive or bait dogs in a confined space; fights end when one dog dies or is cowed or seriously injured	*Aggressors:* Growling, chasing, lunging, biting, wrestling, until one dog is incapable of continuing or is withdrawn. *Victims:* distress calls, retreating/escape attempts, defensive behaviour, appeasement gestures, cowering, trembling	Extreme anger, fear, and/or panic; subsequent extreme pain from serious bite and ripping injuries; potential frustration, helplessness, pain, and discomfort from lasting injury-induced disabilities such as partial blindness or crippling nerve, muscle, tendon and/or bone damage; potential for post-traumatic stress disorder Anxiety and fear may be expressed by survivors in circumstances similar to those in which the cruel act took place, e.g., presence of other dogs
*Extreme restraint:*	Entire body with flexed legs bound tightly with nylon cord or adhesive tape for several days	Futile struggling; binding prevents virtually all movement; breathing impeded; unable to get to water or food	Extreme anxiety and fear at physical helplessness; breathlessness and panic due to restricted breathing; marked thirst and hunger; physical discomfort
*Excessive and prolonged confinement:*	Isolated in coffin-like cage in low-light-intensity room for a prolonged period	Stands up and lies down but cannot turn around; self-grooming severely impeded; no exercise possible; self-mutilated forepaws gnawed to the bone	Extreme loneliness, depression, and anxiety from isolation; boredom and malaise from the barren constancy of the environment; helplessness; frustration and discomfort at inability to groom; severe pain from self-mutilated paws
*Close confinement:*	Continuously kept alone in a small cage or kennel or on a very short tether with inadequate shelter and shade; resting area fouled by urine and faeces	Stereotypical pacing, jumping, and circling but mostly withdrawn and inactive; shivering when cold and panting when hot; self-grooming ineffective; coat unhygienic; suppurating skin sores	Extreme loneliness, depression, and anxiety, plus boredom and frustration in restricted environment; helplessness; thermal discomfort in very cold or hot weather; physical discomfort from hard lying areas; persistent itch and pain from skin sores
*Prolonged tethering*	Kept on a tether outdoors, alongside other dogs	Stereotypical pacing; lunging to the end of the tether; vocalisation; self-mutilation; licking, chewing, and/or swallowing accessible non-nutritive material (e.g., bedding, rocks, tethers, fabric of shelters)	Intense helplessness, depression, and/or frustration at thwarted urges for physical contact with other dogs and for free-running exercise and play; anxiety and fear from inescapable threats; physical discomfort and neck pain from collar-induced lunging injuries
*Brutal tethers:*	A tight collar, chain, rope, or wire used to tether a growing dog where the loop becomes deeply embedded in the neck	Laboured breathing due to tracheal compression; restlessness; frequent, prolonged scratching at the tightening loop and then the infected neck wound	Marked breathlessness and anxiety due to impeded breathing; increasingly severe pain and panic as the neck loop embeds more deeply and the skin breaches exposing raw and infected underlying tissue
* Exposure to severe cold:	Exposure to unrelieved and extreme ambient cold, such as living in cold, wet, and/or windy conditions, or being confined on frozen ground	Seeking warmth, huddling with other animals, curling up to minimise heat loss, alternating which feet are placed onto frozen ground, intense shivering, hypothermic lethargy	Considerable chilling discomfort and misery, ultimately leading to unconsciousness; cold-induced pain
* Exposure to severe heat:	Exposure to unrelieved and extreme radiant and/or ambient heat from sunshine, fires, overheated vehicles and other enclosures, and from contact with hot surfaces	Seeking shade and water; intense panting; ingestion of grass or other materials to alleviate nausea, then reduced panting, and convulsions	Considerable hyperthermic discomfort and pain; nausea; extreme thirst and panic; hyperthermic dizziness, lethargy, and weakness; physical exhaustion
*Abandonment:*	Left confined or discarded with no provision for any necessities of life, e.g., water, food, shelter, shade, variety, company, and care	Distress calls; escape attempts from confinement; fruitlessly seeking companionship; seeking water and food, plus warm or cool, dry, comfortable, and hygienic resting areas; when the dog feels unsafe, hiding and reduced maintenance behaviour; sickness behaviours specific to developing pathologies	Moderate-to-very-severe loneliness, thirst, hunger, hypo- or hyper-thermic distress, physical discomfort, depression, anxiety, fear and/or panic; and, depending on the clinical pathology, breathlessness, pain, nausea, dizziness, debility, weakness, and/or sickness
*Prolonged restraint in a trap:*	Caught in an unmonitored leg-hold jaw or snare trap for several days	Initially extreme and fruitless escape attempts, then inactive unless disturbed by other animals or events; no access to water, food, shelter, shade, or company; self-mutilation to facilitate escape	Extreme pain from the trap; severe anxiety, fear, and panic from inability to escape; physical discomfort; after some days, withdrawal, depression, lethargy, and loneliness compounded by marked-to-very-severe thirst and hunger, as well as potential hypo- or hyper-thermic distress; marked pain from self-mutilation
*Absent or delayed veterinary care:*	No or slow provision of veterinary care for injuries or diseases that require it	Injury-specific and disease-specific behaviours	Potentially any or all of the following: marked-to-very-severe thirst, hunger, pains of different types, breathlessness, nausea, dizziness, debility, weakness, sickness, anxiety, fear, panic, helplessness, loneliness, depression, frustration, and anger
*Exposure to female(s) in oestrus:*	Intact males in breeding facilities in close proximity to females in oestrus	Escape attempts; excessive barking; excessive pacing; excessive licking/chewing of genitals. Can lead to increased aggressive interactions between individuals.	Sexual frustration; anxiety; injury-induced pain from thwarted attempts to access females in oestrus; pain from self-mutilation
*Exposure to social threats without physical harm:*	Interactions with humans that involve yelling, shouting, and other forms of intimidation, as may occur during training and restraint; threatening interactions with other dogs	Defensive behaviour: avoidance, retreating, cowering, trembling, appeasement gestures, tucked tail, distress calls	Anxiety, fear, and/or panic
